# Allele-specific expression: applications in cancer and technical considerations

**DOI:** 10.1016/j.gde.2020.10.007

**Published:** 2021-02

**Authors:** Carla Daniela Robles-Espinoza, Pejman Mohammadi, Ximena Bonilla, Maria Gutierrez-Arcelus

**Affiliations:** 1 Laboratorio Internacional de Investigación sobre el Genoma Humano, Universidad Nacional Autónoma de México, Campus Juriquilla, Boulevard Juriquilla 3001, Santiago de Querétaro 76230, Mexico; 2 Wellcome Sanger Institute, Hinxton, Cambridgeshire CB10 1SA, UK; 3 Department of Integrative Structural and Computational Biology, The Scripps Research Institute, La Jolla, CA, USA; 4 Scripps Translational Science Institute, The Scripps Research Institute, La Jolla, CA, USA; 5 Department of Computer Science, ETH Zurich, Universitätsstr. 6, 8092 Zürich, Switzerland; 6 Swiss Institute of Bioinformatics, Quartier Sorge - Bâtiment Amphipôle, Lausanne 1015, Switzerland; 7 University Hospital Zurich, Rämistrasse 100, 8091 Zürich, Switzerland; 8 Center for Data Sciences, Brigham and Women’s Hospital, Harvard Medical School, Boston, MA 02115, USA; 9 Division of Genetics, Department of Medicine, Brigham and Women’s Hospital, Harvard Medical School, Boston, MA 02115, USA; 10 Division of Rheumatology, Inflammation and Immunity, Department of Medicine, Brigham and Women’s Hospital, Harvard Medical School, Boston, MA 02115, USA; 11 Program in Medical and Population Genetics, Broad Institute, Cambridge, MA 02142, USA; 12 Division of Immunology, Department of Pediatrics, Boston Children's Hospital, Harvard Medical School, Boston, Massachusetts, USA

## Abstract

Allele-specific gene expression can influence disease traits. Non-coding germline genetic variants that alter regulatory elements can cause allele-specific gene expression and contribute to cancer susceptibility. In tumors, both somatic copy number alterations and somatic single nucleotide variants have been shown to lead to allele-specific expression of genes, many of which are considered drivers of tumor growth. Here, we review recent studies revealing the pervasive presence of this phenomenon in cancer susceptibility and progression. Furthermore, we underscore the importance of careful experimental design and computational analysis for accurate allelic expression quantification and avoidance of false positives. Finally, we discuss additional methodological challenges encountered in cancer studies and in the burgeoning field of single-cell transcriptomics.

**Current Opinion in Genetics and Development** 2021, **66**:10–19

This review comes from a themed issue on **Cancer genomics**

Edited by **David J. Adams**, **Marcin Imielinski** and **C. Daniela Robles-Espinoza**

For a complete overview see the Issue and the Editorial

Available online 28th December 2020

**https://doi.org/10.1016/j.gde.2020.10.007**

0959-437X/© 2020 Published by Elsevier Ltd.

## Introduction

**Figure 1:**
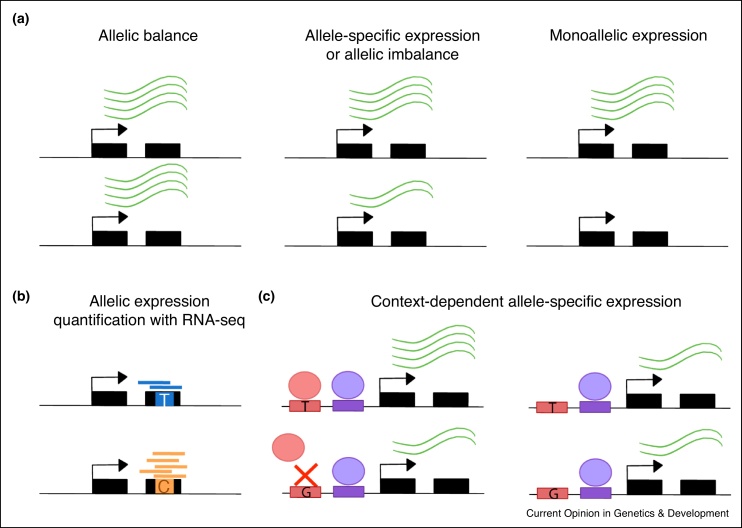
Allele-specific expression. **(a)** Schemes depicting types of allelic expression. Allelic balance, when both gene alleles are equally expressed. Allele-specific expression or allelic imbalance when one allele is significantly more expressed than the other one. Monoallelic expression, when only one gene allele is expressed. **(b)** Scheme illustrating how allelic expression quantification is performed using RNA-seq, by taking advantage of exonic heterozygous sites and counting the number of reads mapping to each allele. **(c)** Scheme illustrating one hypothetical mechanism of how context-dependent allele-specific expression can occur.

The human genome is diploid, with each individual generally carrying two copies of each chromosome. Each chromosome harbors one copy of each gene, referred to as allele, each of which is inherited by one of the two parents. The two gene alleles are generally expressed at similar levels in a tissue, but *cis*-regulatory differences between them, for example, differential binding of transcription factors (TFs), can lead to systematic differences between the expression of the two alleles in an individual (Figure 1a). This is commonly referred to as allelic imbalance or Allele-Specific Expression (ASE). In extreme instances, only one copy of the gene is expressed, a phenomenon called mono-allelic expression, that can be due to gene deletion or to epigenetic mechanisms such as imprinting or X chromosome inactivation. The gene expression measured for each gene allele individually, called from now on allelic expression quantification, can be generated using RNA sequencing data and allows to quantify the degree of allelic imbalance in each gene (Figure 1b) [1,2^•^].

**Figure 2:**
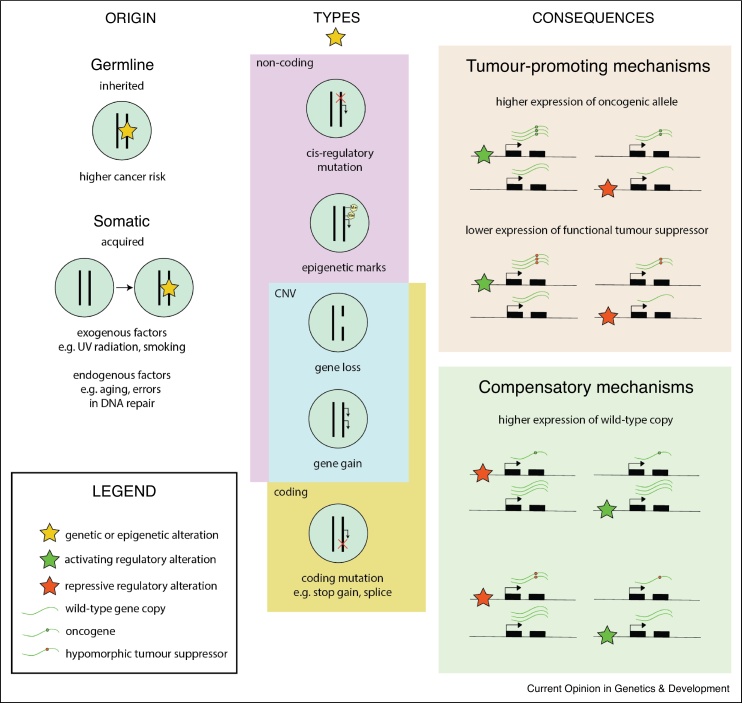
The origins, types, and consequences of ASE in cancer. ASE can have both a germline or somatic origin (Left panel). The former refers to the case where genetic alterations are inherited from the parents, and the latter when these are acquired during the lifetime of the individual. Germline and somatic alterations can be non-coding, when they affect *cis*-regulatory elements or consist of an aberration in epigenetic mark configuration, or coding when stop-gained or splicing mutations lead to nonsense-mediated decay and preferential wild-type allele expression (middle panel). Copy number alterations, including gene gains and losses, can span both coding and non-coding genetic regions. These alterations can result in tumor-promoting mechanisms, such as higher expression of oncogenes or lower expression of functional tumor suppressors, which can then lead to aberrant cell cycle control, impaired DNA repair response, or other tumor-promoting mechanisms. However, they theoretically may also lead to compensatory mechanisms if the wild-type copy is expressed at higher levels than the mutant copies.

In non-cancerous tissues, ASE is driven mostly by genetic regulatory variation in *cis*, specifically by genetic variants that transcriptionally or post-transcriptionally influence the amount of mRNA present from each allele [3, 4, 5]. For example, a genetic variant could affect mRNA decay [6] or alternative splicing [7,8], which can lead to ASE for a gene. In cancer tissues, on the other hand, ASE is often driven by somatic copy number variation (SCNA) of one allele, including focal amplifications of cancer-promoting genes or loss of a wild-type tumor suppressor gene copy, which can often confer a selective advantage to tumor growth [9]. Furthermore, non-coding germline genetic variants can exert their effect on cancer predisposition and progression by causing ASE. These data can be used to study the cis-regulatory footprint of both germline variants and somatic mutations in cancer (Figure 2). Additionally, these allelic expression phenomena can be cell-type or cell-state dependent [10, 11, 12, 13, 14]. For example, TFs driving ASE may be differentially active across cell states, so allelic imbalance may not be observed in all contexts or environments (Figure 1c). This may also influence how and when traits are altered and may therefore lead to disease.

In this Review, we describe how ASE has been shown to influence cancer development and progression, with an emphasis on the most recent discoveries. We then outline how allelic expression can be measured accurately along with its technical and analytical challenges, with a particular emphasis on cancer-associated complexities and the transformative single-cell omics technologies, and we conclude with our view on future perspectives.

## Allele-specific expression in cancer

### Genetic predisposition to cancer

Recently, allelic expression has been used to investigate the target genes of non-coding variants within regions associated with cancer predisposition through genome-wide association studies (GWAS). One of the first ASE-associated loci that was described in colorectal and prostate cancer was rs6983267, which lies in an enhancer region and affects the expression of the well-known *c-MYC* oncogene [15,16], and since then, other studies have fine-mapped variants in other regulatory regions that may influence gene expression in other cancer types [17, 18, 19]. For example, Choi *et al.* focused on a 100 kb region in chromosome 1 that has been associated to melanoma risk, and observed that the tag SNP rs3219090 was associated to ASE in *PARP1*, a gene coding for an enzyme that participates in DNA repair, and fine-mapped this effect to preferential binding of RECQL, another DNA repair protein, to a nearby indel [20]. According to the authors, the risk allele then translates into higher PARP1 levels, which may promote melanoma formation by rescuing cells from *BRAF^V600E^* oncogene-induced senescence. Another example involves intronic cis-acting variants in individuals predisposed to breast and ovarian cancer that lead to haploinsufficiency of the DNA repair proteins PALB2 and BRCA1 [21,22], and regions in the genome with differential promoter/enhancer activity between matched tumor and normal samples, that overlap GWAS-associated variants in renal cell carcinoma [23]. In an instance that exemplifies the complexities of cancer risk and gene regulation, Hua *et al.* identified that prostate cancer risk SNP rs11672691 falls within an intron of lncRNA *PCAT19*, in a region with both promoter and enhancer function. Risk allele G was associated with both a lower expression of the short form of lncRNA *PCAT19* (affecting its promoter function) and a higher expression of its long form (affecting its enhancer function) via decreased binding of transcription factors YY1 and NKX3.1 [24^•^]. This long form then promotes prostate cancer development by cooperating to activate a number of cell-cycle genes. To facilitate performing these analyses systematically, methods such as PLASMA [25] and the statistical framework developed by Zou *et al.* [26] have been introduced.

Furthermore, allele-specific mechanisms have been speculated to play a role in modifying the penetrance of deleterious variants, for example in individuals with Li-Fraumeni syndrome, caused by germline damaging variation in the tumor suppressor gene *TP53* [27]. Buzby *et al.* reported a father-daughter duo where both were heterozygous carriers of a deleterious *TP53* Ser241Tyr variant, but only the daughter developed tumors. Cells from the father showed a significantly higher wild-type/mutant *TP53* ASE ratio than those from the daughter, which allowed him to have comparable total *TP53* expression levels to those of homozygous wild-type *TP53* cells. Although the authors did not investigate the causes of ASE, they speculate that these *TP53* alleles may be subject to imprinting or an undescribed epigenetic regulatory mechanism. This was a targeted search, but generalizing these observations, Castel *et al.* [28^•^] used phased genomes from healthy individuals and cohorts from The Cancer Genome Atlas (TCGA) [29], and discovered that cancer patients had an enrichment of risk-increasing haplotype configurations, consisting of a rare coding variant on a higher-expressed haplotype, compared to controls. This suggests that germline cis-regulatory variants can modify the penetrance of coding variants.

### Somatic mutations leading to allelic imbalance

ASE tends to be more common in tumors as compared to normal tissues, and is largely driven by SCNAs [30, 31, 32]. For example, a recent study characterized the genomic contribution to RNA alterations in the tumor transcriptomes of 1188 patients. The authors reported that SCNAs accounted for 84.3% of the variation in ASE, and germline variants associated with expression levels (eQTL) explained 9.1% [33^••^]. Interestingly, somatic single nucleotide variants with a stop-gain effect leading to nonsense-mediated decay (NMD) composed the most relevant mechanism to explain ASE at an individual level, in line with findings from rare genetic variation in healthy populations in GTEx data [5]. Furthermore, the authors found that ASE driven by somatic variation is enriched in cancer driver genes, suggesting that the allelic effects can act as a driver and are not only a consequence of cancer-associated genomic aberrations. A similar result was observed by Przytycki and Singh, who developed a method to detect differential ASE between normal and tumor samples and, when applied to TCGA breast cancer samples, found that known cancer genes exhibit this phenomenon, with SCNA and NMD being important contributors [34^•^]. Another smaller study focusing on 11 recurrently mutated genes in acute myeloid leukemia found that 9 showed ASE, supporting the idea that ASE may be a common event in cancer [35].

Allelic imbalance can also be studied at the DNA level by quantifying SCNAs, and assessing whether the gene allele carrying a coding somatic mutation was specifically amplified or lost. A study of more than a thousand likely driver mutations across 69 oncogenes in 13 448 tumors concluded that nearly half (45%) showed allelic imbalance in DNA copy numbers, and that 41% of all samples studied across 53 cancer types showed mutant allele imbalance of at least one oncogenic mutation [9]. Focal amplifications, loss of the wild-type allele, and “hitchhiking” (to a lower extent), were all found to play a role in these observations, and distinct mechanisms behind them were described (e.g. tumor suppressor genes were mostly affected by the loss of the WT allele whereas oncogenes showed mainly single-copy genomic gains of the mutant allele). It is not unreasonable then to expect that these genomic aberrations will translate to a transcriptional bias leading to ASE. All these large studies indicate that a significant fraction of driver alterations in cancer are associated with ASE events, and that considering the allelic imbalance state of cancer-associated genes may provide additional prognostic information. These observations further support the involvement of this mechanism in tumor development.

Overall, these studies exemplify the utility of studying ASE and its role in cancer susceptibility and tumor progression. Here, we would like to underscore the importance of applying rigorous methodologies to its study. While ASE has the advantage of comparing expression levels of two alleles subject to the same technical and biological environment, it can easily lead to false positives if technical biases and noise are not considered. Below we highlight the main computational and experimental aspects that need to be taken into account when performing ASE analyses.

## Allelic expression quantification methods and technical considerations

**Figure 3:**
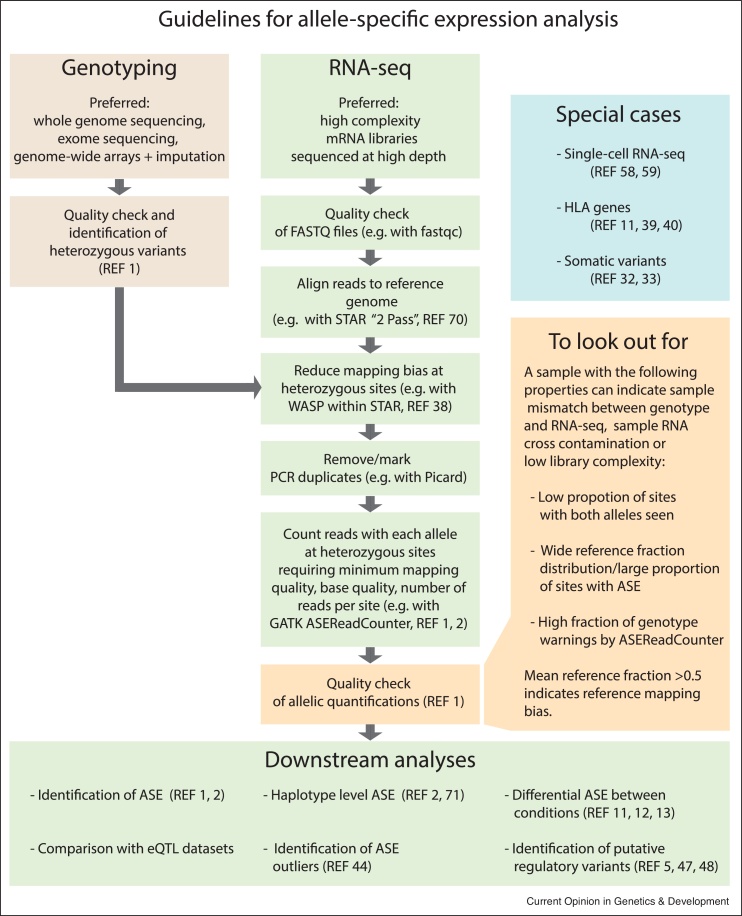
Guidelines for allele-specific expression analysis. Scheme depicting the main steps for allelic expression quantification, quality check, optional downstream analyses to study ASE, and special cases with extra challenges. Recommended tools and publications are cited [70,71].

Allelic expression quantification is typically measured by taking advantage of heterozygous sites inside exons and counting the number of next generation sequencing (NGS) reads mapping over the heterozygous site that displays one allele versus the other (Figure 1b). If the ratio between the two alleles significantly deviates from the expected 50:50, then this locus is deemed to show ASE or allelic imbalance. However, in order to quantify allelic expression in an accurate and reliable way, there are multiple experimental aspects and computational biases to take into account when designing an experiment and analyzing the resulting data. Figure 3 depicts the main steps and recommended guidelines to perform allelic expression analyses. As detailed best practices for ASE analyses are out of the scope of this review, we would like to direct those readers interested in greater depth in the topic to Castel *et al.* [1], as well as the other references cited in Figure 3 and throughout this section.

First, for a given individual, heterozygous variants in genes need to be identified. This can be done by a number of methods, including whole genome sequencing, exome sequencing, genotyping microarrays, and RNA-seq (although extra considerations should be taken if the latter is chosen, as variant calling from RNA-seq has inherent limitations that may significantly impact their reliability in ASE analyses [1]). Then, once RNA-seq is performed on the tissue of interest, and after proper quality check, reads are aligned to the reference genome. Here, mapping bias is an important aspect to control for when quantifying allelic expression. During the alignment of reads to heterozygous sites on the reference genome, reads that contain the reference allele will align better than reads with the alternative allele. This can lead to a higher number of genes with false-positive ASE signal, since the reference allele will be overrepresented [1]. Several strategies have been proposed to alleviate mapping bias in allelic expression data such as using variant-aware aligners [36,37], or discarding reads that would not map uniquely to the same position if their allele is flipped [38]. For highly polymorphic genes, such as Human Leukocyte Antigen (HLA) genes, mapping bias is even more problematic. For example, individuals that have HLA alleles that are highly different from the reference genome can appear to have lower gene expression levels (or lower DNA copy number dosage) than individuals with reference alleles. At present, the best approach to quantify allelic expression on HLA genes is to use a personalized genome for each individual, containing their specific HLA alleles [11,39,40]. Some limitations may soon be overcome by technological advances on long read sequencing approaches applied to HLA allelic expression quantification as well as to isoform-specific allelic expression determination [41,42].

Another aspect to take into account when testing for ASE is the overdispersed nature of allelic expression data, which can cause false positives if assuming a standard binomial distribution [38,43]. To avoid this, extra-binomial variation has been accounted for using beta-binomial models [13,38], binomial-logit-normal distribution [44] or by estimating overdispersion as a random effect in a binomial generalized linear-mixed model [11,12].

Careful experimental design can also ensure better quality of ASE analysis. For example, longer reads are preferable over shorter reads as the latter are significantly more prone to display mapping bias. Additionally, library complexity can influence the quality of ASE data, with low complexity libraries (such as those with low amount of RNA starting material) or libraries with a large amount of low complexity sites (due to low number of reads with unique starting positions for example) posing extra challenges [45,46]. Higher sequencing depth and higher number of heterozygous sites in expressed regions of the genome (usually exonic, and occasionally intronic and intergenic regions) yield higher power to detect allelic imbalance signals. Hence, when comparing the degree of ASE between different samples (such as healthy tissue against tumor), variation in read depth and number of heterozygous variants ascertained should be taken into account. It is also important to keep in mind that while ASE largely reflects the effects of regulatory variants, it does not reveal information about the regulatory variant itself, and further investigation is needed for that purpose [5,47,48].

### Allelic expression quantification in cancer

While allelic expression quantification in cancer follows in general the same steps as the analysis in non-cancer tissues, there are extra challenges that need consideration. For example, SCNAs on the tumor may affect the interpretation of an identified ASE event in an exonic heterozygous germline variant and therefore, copy-number profiles of the tumor and of the matched normal should be integrated into the analysis of a cancer sample if available. Similarly, other somatic variants may affect detection of ASE. For example, small indels produce stronger mapping biases. And if allelic expression is to be measured over exonic somatic single nucleotide variants (SNV), care should be taken as to how these somatic variants are called, since both the fraction of tumor cells in a sample and sequencing depth can impact the number of reads observed for a given gene and each of its alleles [49,50]. There may also be tumor-specific biases to take into account given that tumor mutation burden varies among tumor types and among patients with the same tumor type [51]. Theoretically, a higher tumor mutation burden (TMB) could mean that more somatic variants within genes would be available to detect ASE and/or more regulatory somatic variants could be causing ASE. In a recent study comparing tumor types there does not seem to be a clear correlation between high number of non-synonymous SNVs and high ASE (e.g. melanoma and breast adenocarcinoma [33^••^]). However, it remains to be systematically tested whether different metrics of TMB are associated with higher ASE events, while controlling for read depth. Hence, the somatic or germline nature of the ASE event needs to be properly determined by using both large databases of human genetic variation and a matched normal tissue if available.

### Allele-specific expression in single cells

The latest omic technologies allow us to ascertain the transcriptome of single cells, and theoretically, to detect cell-specific ASE. Single-cell RNA-seq has enabled researchers to assess tumor and immune cell heterogeneity, and discover new cellular states driven by transcriptional programs with important roles in cancer and response to therapy [52, 53, 54]. However, there are still important limitations of this technology. Single-cell RNA-seq protocols are able to capture only a small proportion of the mRNA pool in a cell, which makes the data sparse. Estimates range from 6 to 8% in early high-throughput protocols [55], although with more recent versions it may go up to 32% (10X Genomics URL: https://www.10xgenomics.com/), with low-throughput methods in general having higher sensitivity [56]. For allelic expression quantification, this is a particularly important problem. If only one of the two alleles is detected in a given cell, this does not necessarily mean that the other allele is not expressed; it may just not have been captured by the technique. For example, Borel *et al.* discovered more ASE in single fibroblast cells than in bulk data [57]. While part of this may be biological, it is currently hard to disentangle true allelic imbalance signals from the high levels of technical noise at a single cell level [58,59^•^]. Kim *et al.* proposed to use external RNA spike-ins during library preparation to distinguish technical from biological sources of variation in ASE [58]. A recent study developed a computational method to quantify in a more precise way allelic expression in single cells. This method leverages information from multi-mapping reads and from other cells that are in the same allelic state, which is particularly important in scenarios of low depth sequencing [59^•^]. An additional consideration performing single-cell RNA-seq with the objective of quantifying allelic expression is the area of the gene captured by the specific protocol. For example, protocols that cover the whole transcript, such as Smart-seq3 [60], are able to quantify allelic expression over a higher number of heterozygous sites than protocols that only cover the 3’ or 5’ end of the gene [55].

Despite these technical challenges, allelic expression quantification in single-cell data has already been useful to study a number of biological processes. Groups have quantified allelic expression in the X chromosome to study X chromosome inactivation escape [61,62]. Others have measured allelic expression across phased SNPs within genes to infer transcriptional kinetics in mice, suggesting these are influenced differently by promoters and enhancers [63]. In cancer, allelic information from single-cell RNA-seq data has been used to characterize intra-tumor heterogeneity and identify key transcriptional programs in particular genetic subclones [64,65].

## Conclusions and perspectives

Recent studies have shown that allele-specific mechanisms play a significant role in cancer development. At the germline variant level, several susceptibility variants identified by cancer GWA studies have been shown to have cis-regulatory effects for nearby genes, and there is evidence that this mechanism may also play a role in high-penetrance familial cancer syndromes [27]. At the somatic mutation level, a significant fraction of tumors have at least one somatically acquired-oncogenic mutation displaying allelic imbalance. Furthermore, recent evidence shows that germline regulatory variants can influence tissue immune infiltration [66], response to immunotherapy [67], and gene regulation in tumor-infiltrating lymphocytes only [68]. This suggests that future studies of ASE in both cancer tissues and immune cells may reveal additional insights into mechanisms of cancer development and response to therapy, and potentially even in predicting autoimmunity side effects to treatment [69].

ASE studies allow us to gain insights into important biological processes in gene regulation, and the potential ways in which these events contribute to triggering carcinogenesis, tumor evolution, or response to cancer therapies. However, it is critical to take into account potential technical biases when designing experiments and performing allelic expression bioinformatic and statistical analyses, in order to avoid false positives and derive robust conclusions. This is especially challenging with single-cell experiments, where data are sparse, and cells are often sequenced at low depth. However, as single-cell experimental protocols mature and new computational methods are developed to improve accurate quantification and statistical analysis of allelic expression in single cells, this will deepen our understanding of how ASE changes dynamically between cell states, how it interacts with protein-coding variants, and overall how it influences transcriptional programs that lead to disease or response to therapies. We expect that these developments will contribute to increasing our appreciation of the diversity of mechanisms fueling cancer development.

## Conflict of interest statement

Nothing declared.

## References and recommended reading

Papers of particular interest, published within the period of review, have been highlighted as

• of special interest
•• of outstanding interest
